# Precision Medicine in Childhood Asthma: Omic Studies of Treatment Response

**DOI:** 10.3390/ijms21082908

**Published:** 2020-04-21

**Authors:** Javier Perez-Garcia, Esther Herrera-Luis, Fabian Lorenzo-Diaz, Mario González, Olaia Sardón, Jesús Villar, Maria Pino-Yanes

**Affiliations:** 1 Genomics and Health Group, Department of Biochemistry, Microbiology, Cell Biology and Genetics, Universidad de La Laguna, 38200 San Cristóbal de La Laguna, Spain; jpegarci@ull.edu.es (J.P.-G.); eherrerl@ull.edu.es (E.H.-L.); florenzo@ull.edu.es (F.L.-D.); 2 Instituto Universitario de Enfermedades Tropicales y Salud Pública de Canarias (IUETSPC), Universidad de La Laguna, 38200 San Cristóbal de La Laguna, Spain; mgonzalc@ull.edu.es; 3 Department of Biochemistry, Microbiology, Cell Biology and Genetics, Universidad de La Laguna, 38200 San Cristóbal de La Laguna, Spain; 4 Division of Pediatric Respiratory Medicine, Hospital Universitario Donostia, 20014 San Sebastián, Spain; olaia.sardonprado@osakidetza.eus; 5 Department of Pediatrics, University of the Basque Country (UPV/EHU), 20014 San Sebastián, Spain; 6 Centro de Investigación Biomédica en Red de Enfermedades Respiratorias (CIBERES), Instituto de Salud Carlos III, 28029 Madrid, Spain; jesus.villar54@gmail.com; 7 Multidisciplinary Organ Dysfunction Evaluation Research Network, Research Unit, Hospital Universitario Dr Negrín, 35019 Las Palmas de Gran Canaria, Spain; 8 Keenan Research Center for Biomedical Science at the Li Ka Shing Knowledge Institute, St Michael’s Hospital, Toronto, ON M5B 1W8, Canada; 9 Instituto de Tecnologías Biomédicas (ITB), Universidad de La Laguna, 38200 San Cristóbal de La Laguna, Spain

**Keywords:** genomics, transcriptomics, epigenomics, metabolomics, microbiome, multiomics, SABA, ICS, LTRA

## Abstract

Asthma is a heterogeneous and multifactorial respiratory disease with an important impact on childhood. Difficult-to-treat asthma is not uncommon among children, and it causes a high burden to the patient, caregivers, and society. This review aims to summarize the recent findings on pediatric asthma treatment response revealed by different omic approaches conducted in 2018–2019. A total of 13 studies were performed during this period to assess the role of genomics, epigenomics, transcriptomics, metabolomics, and the microbiome in the response to short-acting beta agonists, inhaled corticosteroids, and leukotriene receptor antagonists. These studies have identified novel associations of genetic markers, epigenetic modifications, metabolites, bacteria, and molecular mechanisms involved in asthma treatment response. This knowledge will allow us establishing molecular biomarkers that could be integrated with clinical information to improve the management of children with asthma.

## 1. Introduction

Asthma is the most prevalent chronic disease in children and youth [1]. Globally, its prevalence reaches 11.7% and 14.1% in children aged 6–7 and 13–14 years old, respectively [2]. Chronic respiratory symptoms of asthma are also remarkably common among children and young adults. Indeed, the pediatric prevalence of recent wheeze exceeds 20% in different regions of Europe, North America, Australia, and Latin America [3]. Additionally, the prevalence of severe asthma, defined by night asthma symptoms as well as the frequency of severe wheezing episodes, surpasses 7.5% in many regions throughout the world [3]. Moreover, the burden of asthma, measured as disability-adjusted life years, reaches one of the highest values in 10–14-year-old children, highlighting the impact of asthma on the quality of life of children [1]. Besides that, asthma also has an outstanding economic burden that increases with disease severity [4].

International guidelines for the management of pediatric asthma based on clinical factors have been established in order to control the symptoms and to reduce the risk of future associated complications, with pharmacological treatment as a key role in achieving asthma control [5]. Reliever medication aims to discharge the symptoms during worsening episodes of the disease known as exacerbations [5]. Short-acting beta-agonists (SABAs) are one of the most common reliever medications in children due to rapid-onset bronchodilation mediated by the activation of β_2_ adrenergic receptors [5,6]. When SABA monotherapy is insufficient to prevent symptoms, controller medication is recommended to reduce airway inflammation, improve the control of the symptoms, and prevent exacerbations and impaired lung function [5]. The most commonly used controller medications are inhaled corticosteroids (ICSs), which have an anti-inflammatory and immunosuppressive effect on lung tissue by interacting with the glucocorticoid receptor [5,7]. As asthma severity increases, the ICS dose may be increased or combined with other controller therapies, such as long-acting beta-agonists (LABAs) or leukotriene receptor antagonists (LTRAs) [5]. While LABAs share the mechanism of action of SABAs, their chemical structure favors long-onset bronchodilation [6]. LTRAs cause both bronchodilation and an anti-inflammatory response as a result of the antagonism effect on the leukotriene receptor [8]. Children with severe asthma require add-on therapies such as oral corticosteroids (OCSs), which are similar to ICSs, except for the systemic effects derived from oral administration. As a consequence, the incidence of adverse reactions increases with OCS treatment, which limits its application to the most severe cases [5,9].

Despite following asthma management guidelines, some patients still have sustained symptoms even under treatment with high-dose ICSs or OCSs [10]. Personalized medicine has recently emerged aimed to select the most appropriate therapy for each patient based on identifying different asthma phenotypes and endotypes [11]. Several clinical procedures are used for this purpose, such as induced sputum and bronchial brushing to characterize atopic asthma [11] or nasal cytology to identify a rhinosinusitis phenotype related to asthma and aspirin sensitivity [12]. However, given the different mechanisms underlying asthma and treatment response, guidelines based only on clinical features are likely limit the success of the treatment [13]. In the last few decades, the development of molecular techniques has led to high-throughput analyses at different biological layers known as omics [13]. The integration of omics data with clinical features and laboratory parameters contributes to a better definition of asthma endotypes and therefore, to select the most appropriate therapy [13]. Moreover, the integration of multiple omics approaches in childhood asthma has revealed new disease mechanisms and has arisen as a viable option moving forward for precision medicine [14,15].

In the last year, the role of different omics in asthma treatment response has been recapitulated in different reviews [16,17,18,19,20,21]. This review aims to provide an update on the latest findings in omic studies of pediatric asthma treatment response. A literature search using different combinations of keywords was therefore conducted using PubMed (Table S1). Studies were eligible if they met the following inclusion criteria: (1) omic studies of treatment response focused on children and youth with asthma, (2) publication date between 1 January 2018 and 31 December 2019, and (3) studies written in English. The criteria to exclude studies were: (1) lack of assessment of asthma treatment response, (2) publications focused on individuals without asthma, (3) studies that did not apply omic approaches, (4) studies focused on animal models, and (5) manuscripts reporting literature reviews, editorials, or opinion articles. A three-stage screening was performed to select eligible studies based on the adequacy of (1) the title, (2) the abstract, and (3) the full text of the manuscripts. All the manuscripts were reviewed by at least three independent authors.

## 2. Pharmacogenomics

Pharmacogenomics involves the study of genetic variation across the genome and its role in treatment response. Most genetic studies of complex diseases like asthma have focused on single nucleotide polymorphisms (SNPs). An SNP involves the variation of one nucleotide in the DNA sequence with a population frequency higher than 1%. Due to linkage disequilibrium (LD) and coinheritance patterns of the polymorphisms, millions of genetic variants can be inferred from genotyping arrays of hundreds of thousands of SNPs. Thus, genome-wide genetic variation can be studied without any prior hypothesis by means of genome-wide association studies (GWAS) [22]. In the last few years, whole-genome sequencing (WGS)emerged as a high-resolution method to study both common and rare genetic variation. Although the WGS approach detects genetic variation not tackled by genome-wide genotyping arrays, its use is still largely limited due to economic constraints [23]. 

In contrast to the past, when most pharmacogenomic studies of childhood asthma were focused on European-descent populations [16,17], within the reviewed period, the pharmacogenomic studies of childhood asthma have analyzed two underrepresented populations with a high burden of asthma and failure in treatment response [24]: African Americans from the Study of African Americans, Asthma, Genes and Environments (SAGE) and Hispanic/Latinos from the Genes-Environment and Admixture in Latino Americans (GALA II) (Table 1, Table S2). In these two studies (GALA II and SAGE), genomic studies of SABA treatment response were carried out by means of GWAS, based on both genotyping arrays and WGS data [25,26], and also by performing admixture mapping [26]. The latter approach is a type of analysis that can be applied to admixed populations in order to identify genomic regions in which local ancestry is associated with a trait, based on the differences in allele frequency of the SNPs depending on their ancestral background [24]. These studies focused on the change in lung function due to SABA administration, which is known as bronchodilator drug response (BDR) [25,26], and also on ICS response, analyzing as an outcome the presence/absence of severe asthma exacerbations [27].

In the first study, Spear et al. [26] conducted a GWAS of BDR in 949 African Americans with asthma from SAGE using genotyped and imputed data. The following population-specific genome-wide significant association of the SNP rs73650726 was found on 9q21 (β ± standard error [SE] for the A allele: −3.8 ± 0.66, *p*-value = 7.69 × 10^−9^). Interestingly, according to the 1000 Genomes Project data, this SNP is only present in African-admixed populations at approximately 9% frequency, but not in Europeans or Asians. Moreover, a trans-ethnic meta-GWAS across 2779 African American and Hispanic/Latino children and young adults identified genome-wide association of three SNPs with SABA response—rs7903366 (β ± SE for the T allele: 1.23 ± 0.22, *p*-value = 3.94 × 10^−8^); rs7070958 (β ± SE for the A allele: −1.24 ± 0.23, *p*-value = 4.09 × 10^−8^); and rs7081864 (β ± SE for the A allele: 1.23 ± 0.22, *p*-value = 4.94 × 10^−8^). These SNPs, which are almost in complete linkage disequilibrium (*r^2^* > 0.95), are located in the protein kinase cGMP-dependent 1 (*PRKG1*) gene and act as expression quantitative trait loci (eQTL) of the *PRKG1* gene in lung tissue. Interestingly, PRKG1 is involved in the nitric oxide/cGMP signaling pathway, participates in the relaxation of smooth muscle, and acts as a key modulator of airway inflammation in response to SABA [28,29]. Moreover, *PRKG1* has been previously associated with lung function and asthma susceptibility [30,31]. Nonetheless, neither the specific-population nor the shared-population SNPs replicated in independent African American and Hispanic-Latino studies. Additionally, an admixture mapping analysis failed to reveal any genomic regions where local ancestry was associated with BDR [26].

Another study also analyzed the same study populations, carrying out WGS on a subset of 1441 patients with asthma that represented the extreme values of the distribution of BDR among African Americans, Puerto Ricans, and Mexicans [25]. While no genome-wide significant association for BDR was found in the specific-population analyses, two SNPs near *DNAH5* were significantly associated with BDR in a trans-ethnic meta-analysis—rs17834628 (OR for the A allele: 1.67, 95% confidence interval [CI]: 1.29–2.16, *p*-value = 1.18 × 10^−8^) and rs35661809 (OR for the G allele: 1.59, 95% CI: 1.20–2.10, *p*-value = 3.33 × 10^−8^). *DNAH5* encodes a protein with ATPase activity, which is involved in a protein complex associated with the microtubules. Remarkably, this gene is involved in allergic sensitization, lung function, and immunoglobulin E (IgE) serum levels [32,33,34]. Besides, the combined effect of common and rare variants in three specific-population loci (1p13.2 and 11p14.1 in Mexicans and 19p13.2 in African Americans) showed a genome-wide significant association for BDR. Moreover, two shared-population loci (4q13.3 and 8q22.1) were significantly associated with SABA response as well.

The third study performed on the GALA II and SAGE studies focused on ICS response and included a subset of 1347 Hispanic/Latino and African American individuals treated with ICSs, which were combined in a meta-GWAS. In this study, the presence/absence of asthma exacerbations was analyzed as a proxy of ICS response [27] (Table 1, Table S2). Asthma exacerbations were defined by emergency room (ER) visits, hospitalizations, or OCS use due to asthma symptoms in the last 12 months while the patient was treated with ICSs. A suggestive association was found for the SNP rs5995653 from the intergenic region *APOBEC3B-APOBEC3C,* which showed evidence of replication in 1697 European children with asthma (OR for the A allele: 0.76, 95% CI: 0.62–0.93, *p*-value = 7.52 × 10^−3^). Although this SNP did not reach the genome-wide significance threshold in a meta-analysis across all populations (OR for the A allele: 0.70, 95% CI: 0.61–0.81, *p*-value = 3.31 × 10^−7^), the A allele was consistently associated with better ICS response measured as the change in the forced expiratory volume in the first second (FEV_1_) after six weeks of ICS treatment (OR: 2.16, 95% CI: 1.26–3.70, *p*-value = 4.91 × 10^−3^). *APOBEC3B* and *APOBEC3C*, genes that have not been previously associated with asthma, encode subunits of a cytidine deaminase, a protein with an RNA editing function that has an important role in the immune response to several viruses by restricting their replication. Moreover, Hernandez-Pacheco et al. [27] carried out replication analyses of the genomic regions associated with ICS response in prior GWAS focused on Europeans and Asians. The SNP rs62081416 near *L3MBTL4*-*ARHGAP28* was found to be associated with ICS response in African-admixed children (OR for the A allele: 2.44, 95% CI: 1.63–3.65, *p*-value = 1.57 × 10^−5^). Remarkably, both *L3MBTL4* and *ARHGAP28* have been associated with post-bronchodilator lung function [32].

## 3. Epigenomics

Epigenetics involves the study of the mechanisms that regulate gene expression without modifying the DNA sequence, including DNA methylation (DNAm), microRNA (miRNA) regulation, and histone modifications. These mechanisms are heavily affected by environmental exposures and are essential for cell differentiation processes. Today, epigenetic changes can be analyzed through the whole genome (i.e., epigenomics) by means of high-throughput techniques. The most studied field in epigenetics is DNAm patterns, which consist of the methylation of a cytosine base that occurs at higher frequencies in regions where the cytosine is followed by a guanine in the 5′-3′ direction (CpG sites) [35,36,37]. 

During the reviewed period, two epigenome-wide association studies (EWAS) conducted by Wang et al. analyzed the association of CpG sites methylation status with treatment response in childhood asthma (Table 2, Table S3). Both evaluated the effects of ICS response on DNA methylation patterns in peripheral blood cells (PBCs) [38,39]. In the first study [38], treatment response was measured by the following two outcomes: (1) the absence of severe asthma exacerbations defined by ER visits or hospitalizations and (2) the absence of OCS use, both of them related to asthma symptoms in the last year despite ICS therapy. A relative hypomethylation of the CpG site cg00066816 near *IL12B* showed a protective effect for severe exacerbations after false discovery rate (FDR) adjustment (*q*-value = 0.028) in a meta-analysis across non-Hispanic whites from the Childhood Asthma Management Program (CAMP, n = 154), Europeans from the Children, Allergy, Milieu, Stockholm, Epidemiology (BAMSE, n = 72), and Hispanic/Latinos from the Genetic Epidemiology of Asthma in Costa Rica Study (GACRS, n = 168). Hypomethylation of cg00066816 and the absence of severe exacerbations was shown to be specific to patients treated with ICSs, since it was not observed in European children treated with placebo (standardized coefficient: −3.051, *p*-value = 0.002). Additionally, hypomethylation of cg00066816 was associated with lower *IL12B* expression in blood cells in Europeans (Pearson coefficient [*ρ*] = 0.34, *p*-value = 0.01), although this result was not confirmed in non-Hispanic white children from CAMP. *IL12B* encodes a subunit of two cytokines (IL-12 and IL-23) involved in the immune response and airway hyperresponsiveness, whose expression levels have been related to the response to corticosteroids in bronchial biopsies from asthma patients [40,41,42].

Moreover, in the same study, Wang et al. found 13 CpG sites that were significantly associated with the absence of OCS use (*q*-value < 0.05) in a meta-analysis across non-Hispanic whites from CAMP and Costa Ricans from GACRS (n = 322). An interaction analysis identified that hypermethylation of cg04256470 near *CORT-CENPS* was associated with the absence of OCS use specifically in patients treated with ICSs (standardized coefficient: 2.322, *p*-value = 0.02). Interestingly, relative hypermethylation of cg04256470 was associated with higher *CORT* expression in CAMP (*ρ* = 0.2, *p*-value = 0.045) [40,41,42]. *CORT* encodes the peptide cortistatin, which has a role in the anti-inflammatory process through the hypothalamic-pituitary-adrenal axis and regulates endogenous corticosteroids [43,44].

The second EWAS of ICS response analyzed the change in FEV_1_ after eight weeks with ICS treatment in non-Hispanic white children from CAMP (n = 152) [39]. Relative hypermethylation of cg27254601 from *BOLA2* was associated with lung function improvement (standardized coefficient: 3.598, *p*-value = 0.0005) and with an increased expression of *BOLA2* (*ρ* = 0.25, *p*-value = 0.02). *BOLA2* encodes a protein involved in the maturation process of iron-sulfur containing proteins [45]. Gene expression levels of *BOLA2* in airway cells differ from patients with asthma and healthy individuals [43], and some intronic variants were associated with eosinophil levels and lung function [31,46,47]. Furthermore, hypermethylation in PBCs of the *OTX2* gene, previously found to be hypomethylated in nasal cells from good OCS responders [48], was found to be nominally associated with an improvement in FEV_1_ after ICS treatment (standardized coefficient for cg15607672: 2.123, *p*-value = 0.036). *OTX2* encodes a transcription factor that mainly acts in nervous tissue.

Of note, only one study has performed miRNA profiling to investigate its association with treatment response without prior hypothesis. Kho et al. evaluated the role of 754 circulating miRNAs on the use of OCSs in the past 12 months in serum samples from non-Hispanic white children with asthma from CAMP before initiating an ICS treatment (n = 153) [49]. From the 125 miRNAs that remained after quality control, a total of 12 miRNAs were associated with the risk of exacerbations, defined as the need for more than one steroid burst in the past year because of asthma despite being treated with ICSs (Table 3, Table S4). The miR-206 showed the strongest association, with its serum expression levels being higher in non-exacerbators compared to exacerbators (OR: 0.60, 95% CI: 0.42–0.83, *p*-value = 0.004). Moreover, these miRNAs were included in a predictive model for asthma exacerbations. A combined model based on clinical features and three of these circulating miRNAs levels (miR-206, miR-146b-5p, and miR-720) better predicted asthma exacerbations in children treated with ICSs (area under the receiver characteristics curve [AUC] = 0.81) than a model that only included clinical parameters (AUC = 0.67). Interestingly, these three miRNAs have been related to asthma physiopathology in cell and animal model studies [49] and a previous study associated two of them—miR-146b-5b and miR-206—with baseline FEV_1_/FVC [50]. Moreover, this study revealed four biological pathways regulated by the three miRNAs, which included two that had been previously related to asthma: “inactivation of GSK3 by AKT causes accumulation of b-catenin in alveolar macrophages” (*q*-value = 0.017) and “NF-κβ signaling pathway” (*q*-value = 0.08) [49].

## 4. Transcriptomics

Transcriptomics is the study of the set of all RNA transcripts by high-throughput methods such as RNA sequencing (RNA-seq) or microarrays. Within the reviewed period, the vast majority of the transcriptomic studies of treatment response in pediatric asthma focused on ICSs. A microarray analysis of peripheral blood mononuclear cells (PBMCs) from Taiwanese children with asthma revealed that patients with poor asthma control show specific transcriptomic patterns associated with glucocorticoid signaling and immune response when compared to other children with asthma [51]. Moreover, two studies applied system biology approaches to investigate transcriptomics of ICS response in non-Hispanic white children from CAMP. Qui et al. [52] analyzed gene expression networks in immortalized B-cell lines from 145 children treated with ICSs. Subjects were classified as good (n = 47) or poor (n = 48) ICS responders based on changes in post-FEV_1_% after being treated with ICSs for two months. Good responders showed enrichment in immune response and proapotosis corticosteroid-induced pathways, whereas poor responders had an enrichment in antiapoptosis pathways. Two transcription factors (TFs), *NFKB1* and *JUN,* showed remarkable differential regulation between both groups. The effect of these TFs on the expression of nine downstream genes was evaluated by TF silencing. *CEBPD* (regulated by *NFKB1*) was overexpressed in good responders compared to poor responders while *TMEM53* (regulated by *JUN*) showed the opposite effect. Nonetheless, the lack of validation of the other downstream genes might be because this assay was performed only in a reduced number of subjects and other TFs may simultaneously coregulate the expression of these genes. *NFKB1* encodes a subunit of a transcription regulator (NFKB) of multiple biological pathways. Dysregulation of *NFKB1* was associated with inflammatory diseases and inadequate immune cell development. *JUN* encodes a protein that regulates gene expression by directly interacting with the DNA sequence and also has been related to macrophage activation [53]. 

McGeachie et al. [54] conducted a multiomic analysis in 104 non-Hispanic white children with asthma from CAMP treated with budesonide. Treatment response was evaluated by the steroid responsiveness endophenotype (SRE), a composite phenotype that predicted ICS responsiveness. The SRE index, genome-wide genotyping data, and the response of immortalized lymphoblastoid cells to dexamethasone were integrated to build a steroid response network. A total of seven genes associated with steroid response were identified by this system biology approach, and four of them were selected for in vitro validation analysis. The knockdown of one of these genes, *FAM129A*, reduced dexamethasone response (*p*-value < 0.001) in lung epithelial cells. Interestingly, this gene encodes for a protein involved in an apoptosis pathway [55]. Thereby, *FAM129A* could enhance the anti-inflammatory effect of ICSs.

Katayama et al. [56] performed the only transcriptomic study that evaluated LTRA response in children. A total of 107 children aged 6–48 months were recruited during an acute wheezing episode and were followed-up to seven years. A weighted gene co-expression network analysis (WGCNA) [57] was performed to identify subsets of heavily correlated genes (denominated modules) involved in LTRA response. The WGCNA of gene expression in leucocytes identified a module of 145 co-regulated genes correlated with acute wheezing. This module was enriched in genes involved in interferon signaling pathways, inflammation, and antiviral response. Moreover, this module showed a positive correlation with lung function and LTRA treatment, as well as a negative correlation with vitamin D levels at seven years and with the number of exacerbations during the follow-up period. This module also predicted future LTRA medication with an AUC of 0.81 (95% CI: 0.67–0.96). The gene with the strongest association with LTRA treatment was *TRIM22* (*p*-value = 4.91 × 10^−3^), which encodes a protein involved in antiviral response that is regulated by an interferon pathway [58]. 

## 5. Metabolomics

Metabolomics aims to profile the whole metabolite composition (metabolome) in biological samples. High-throughput analytical techniques, such as mass spectrophotometry, nuclear magnetic resonance, or spectroscopic methods, allow the characterizing of metabolites in invasive samples like blood and in non-invasive ones such as exhaled air (breathomics). This recent omic has been successfully applied for the profiling of asthma [59,60,61] and may contribute to understanding asthma treatment response.

Kelly et al. aimed to evaluate the interaction of age and 501 serum metabolites on BDR after albuterol administration [62]. Blood samples were obtained from children with asthma from CAMP at three time points, with mean ages of 8.8 (n = 560), 12.8 (n = 563), and 16.8 (n = 295), respectively. A total of 39 metabolites, mainly lipids, showed a nominal interaction with age on BDR, being the strongest interaction observed for the 2-hydroxyglutarate (β = −0.004, *p*-value = 1.77 × 10^−4^). Results were evaluated for replication in 320 Hispanic children with asthma from GACRS, with a mean age of 9.1 years. In this case, 12 of 615 metabolites showed a significant interaction with age on BDR, also including 2-hydroxyglutarate (β = −0.015, *p*-value = 0.018). However, the results of the 2-hydroxyglutarate did not survive after multiple comparison adjustments both in CAMP (*q*-value = 0.089) and GARS *(q*-value = 0.997).

Moreover, Kelly et al. also conducted a multiomic study of lung function in Hispanic/Latino children with asthma (n = 325) [63]. In an integrative approach to identify modules of coregulated gene transcripts and metabolites, a total of 25,060 transcripts and 8185 metabolites from whole blood were clustered using WGCNA. Four transcript modules and five metabolite clusters were found to be related to lung function after adjustments for confounders (*p*-value ≤ 0.05) and interactions among seven of them were found. Interestingly, one transcriptomic module, enriched in asthma-related miRNAs, was associated with BDR, and also with a lipid metabolomic module. *ORMDL3*, a gene extensively studied in pediatric asthma that has a role in sphingolipid biosynthesis [64,65,66], was identified as a hub gene of this transcriptomic module. Based on genotype data, the SNP rs8079416 within *ORMDL3* was found to be an eQTL of *ORMDL3* in this population (*p*-value = 6 × 10^−4^) and was also associated with 165 of the 537 lipids included in the metabolomic module. These findings were followed up for replication in 207 children with asthma from CAMP. Both the association of the miRNAs module with BDR (*p*-value = 0.027) and the role of rs8079416 as an eQTL of *ORMDL3* (*p*-value = 5.2 × 10^−10^) were validated. Therefore, the relationship between *ORMDL3* expression, microRNA regulatory motif, and sphingolipid metabolism likely have a role in BDR in pediatric asthma. 

## 6. Microbiome

The composition of the microbial communities, or microbiota, is conditioned by both intrinsic and extrinsic factors [67,68,69]. Microbial exposure is essential to the development of the immune system, and differential changes in the microbiota have been associated with allergic diseases [70,71]. Indeed, dysbiosis in microbial communities and the presence of bacterial pathogens in different body sites (e.g., lung, gut, or tonsils) have been related to the development of allergic diseases, likely due to dysregulation of the host immune response [72,73]. The development of next-generation sequencing (NGS) techniques has allowed characterizing the microbiota from its genetic make-up, known as the microbiome. The diversity and abundance of the microbiome can be assessed by targeted sequencing techniques or metagenomic approaches. To date, the bacterial microbiome has been extensively studied by targeted sequencing of the 16S ribosomal RNA gene (16S rRNA), a prokaryotic marker whose hypervariable regions contribute to the taxonomic classification of bacteria at genus level [74,75].

A longitudinal study conducted by Zhou et al. [76] aimed to identify changes in nasal microbiota related to the risk of asthma exacerbations despite ICS therapy. The nasal bacterial microbiome was characterized by means of 16S rRNA sequencing in 214 European children with mild–moderate persistent asthma treated with low doses of ICSs as part of a clinical trial. Nasal swabs were collected at the time of well-controlled asthma (randomization) and during the first loss-of-asthma-control episode. Children with a nasal microbiome dominated by *Corynebacterium* and *Dolosigranulum* at the time of well-controlled asthma had fewer episodes of early loss of asthma control (*p*-value = 0.005) and longer times to develop at least two episodes (*p*-value = 0.03) when compared to those children with a nasal microbiota dominated by *Staphylococcus*, *Streptococcus*, or *Moraxella*. Moreover, during the first loss of asthma control episode, *Streptococcus* became the most prevalent dominant genus in the nasal microbiome (*p*-value = 0.001). Additionally, bacterial richness and total bacterial load were significantly higher during asthma control loss than in the well-controlled time point (*p*-value = 4 × 10^−4^ and *p*-value = 4 × 10^−5^, respectively). Furthermore, a higher relative abundance of *Corynebacterium* was associated with a lower risk of suffering from asthma exacerbations requiring OCS use (OR: 0.92, 95% CI: 0.89–0.94, *p*-value = 0.04). Finally, the switch of dominant genera from *Corynebacterium* + *Dolosigranulum* to *Moraxella* was associated with the highest risk of OCS use (*p*-value_chisq-test_ = 0.04). *Corynebacterium* is the most abundant commensal bacteria in the nasal microbiome from healthy individuals, and its relative abundance is decreased in patients with asthma [77,78]. Moreover, *Moraxella*, *Streptococcus*, and *Haemophilus* are bacterial pathogens more common in the nasal microbiome of patients with asthma compared to healthy individuals [78,79,80], and *Moraxella* has also been associated with a higher risk of asthma exacerbations [81].

## 7. Discussion

Between 2018 and 2019, a total of 13 studies of different omics have investigated asthma treatment response in children. In detail, three pharmacogenomic, three epigenomic, three transcriptomic, one metabolomic, one microbiome, and two integrative omic studies evaluated the responsiveness to SABAs, ICSs, or LTRAs in children with asthma. These pharmacological therapies are the most commonly used therapies in the daily management of childhood asthma. However, due to the increase of biological medications as add-on therapies in severe asthma, further studies are necessary to understand the mechanisms underlying treatment response of these novel treatments.

Remarkably, in recent years, the number of pharmacogenomic, epigenomic, and metabolomic studies that included ethnically diverse minority populations such as African Americans and Hispanic/Latinos have increased compared to the past [25,26,27,38,63,82,83]. However, studies in populations of Asian ancestry remain still scarce [51]. To move toward precision medicine, further efforts need to be made by the research community in establishing international collaborations with an ethical racial/ethnic representation. Indeed, pharmacogenomic studies can benefit from performing trans-ethnic meta-analysis, since this approach improves signal detection by comparing the effects on different LD structures across populations of diverse ancestries. 

While single omics have contributed to revealing some insights on the basis of treatment response, in many cases, they focus on single-marker approaches, limiting its predictive power and clinic transferability. As an example of success, system biology has been applied to transcriptomics [52] and metabolomics [63] data to identify multiple co-expressed markers and the underlying biologic pathways they are involved in. Further validation should also be sought by ex-vivo studies and directed to the development of panels of biomarkers with clinical applicability. Moreover, many studies have investigated whole tissues with a heterogeneous composition of multiple cell types [38,39,49,51,63], but more initiatives to refine specific cell-type patterns in disease-relevant tissues are required. Besides, while replication attempts are a common practice among pharmacogenomic studies, this strategy should expand to other omics, especially when sample sizes are limited. Furthermore, different definitions of treatment response have been considered across studies, such as BDR, asthma exacerbations, change in FEV_1_ after ICS treatment, asthma control, or a composite measurement of several variables. While these definitions may reflect different underlying mechanisms involved in the response to asthma therapies, the lack of more homogeneous definitions limits the comparisons of the findings across studies.

Notably, proteomic and metagenomic studies of treatment response have been scarcely investigated. Regarding the bacterial microbiome, the resolution at species-level is yet to be achieved by sequencing the whole 16S rRNA gene. Additionally, the contribution of the virome and mycobiome to asthma treatment response remains unexplored. This could be examined by the application of metagenomic studies performing shotgun sequencing. Additionally, since the lung microbiome is difficult to sample, the nasal, the salivary, or even the gut microbiome could be examined as non-invasive alternatives to the lung microbiome due to the relationship between the upper and the lower airways [84,85] and the gut-lung axis [72]. Although proteomics and breathomics have been used for profiling of asthma [60,86,87], during the reviewed period, no study has been focused on treatment response in children with asthma. Breathomics and sputum or salivary proteomics could be straightforwardly applicable in the clinics in terms of non-invasiveness, which provides an outstanding benefit in the management of pediatric asthma.

In conclusion, while omics studies have provided insight into the biological mechanisms involved in treatment response, further efforts are required to refine predictive markers with clinical relevance. The integration of clinical features with multiple omic data will be promising in the management of pediatric asthma in order to provide markers that improve the quality of life of children with asthma.

## Figures and Tables

**Table 1:** Summary of the main recent findings of pharmacogenomic studies in childhood asthma.

rsID/ Chr. Band	Chr:Position ^a^	Gene/ Nearest Gene	Effect Allele	Effect	*p*-Value	Reference
**SABA response**						
rs73650726	9:85152666	9q21	A	β = −3.8	7.69 × 10^−9^	[26]
rs7903366	10:53689774	*PRKG1*	T	β = 1.23	3.94 × 10^−8^	
rs7070958	10:53691116	*PRKG1*	A	β = −1.24	4.09 × 10^−8^	
rs7081864	10:53690331	*PRKG1*	A	β = 1.23	4.94 × 10^−8^	
rs17834628	5:12978566	*LINC01194, LINC02220, DNAH5*	A	OR = 1.67	1.18 × 10^−8^	
rs35661809	5:12968341	*LINC01194, LINC02220, DNAH5*	G	OR = 1.59	3.33 × 10^−8^	
1p13.2	1:114177000-1:114178000	*MAGI3, PHTF1, RSBN1*	NA	NA	4.40 × 10^−9^	[25]
11p14.1	11:27507000-11:27508000	*LOC105376671, LGR4, LIN7C*	NA	NA	6.59 × 10^−9^	
19p13.2	19:10424000-19:10425000	*ZGLP1, ICAM5, FDX1L, RAVER1*	NA	NA	3.12 × 10^−11^	
4q13.3	4:73478000-4:73479000	*ADAMTS3, COX18*	NA	NA	6.25 × 10^−8^	
8q22.1	8:97926000-8:97927000	*SDC2, CPQ, LOC101927066, TSPYL5*	NA	NA	1.32 × 10^−7^	
**ICS response**						
rs5995653	22:39404249	*APOBEC3B-APOBEC3C*	A	OR = 0.70	3.31 × 10^−7^	[27]
rs62081416	18:6605442	*L3MBTL4-ARHGAP28*	A	OR = 2.44	1.57 × 10^−5^	

^a^ Position based on GRCh37/hg19 build. rsID: reference SNP cluster ID; Chr: chromosome; SABA: short-acting beta agonist; ICS: inhaled corticosteroid; OR: odds ratio; NA: not available.

**Table 2:** Summary of the main recent findings of epigenomic studies focused on DNAm in childhood asthma.

CpG	Chromosome:Position ^a^	Gene/Nearest Gene	β	*p*-Value	Reference
**ICS response**					
cg00066816	5:158758353	*IL12B*	−3.101	0.002	[38]
cg00557354	13:111767899	*ARHGEF7*	−3.490	0.001	
cg04256470	1:10510465	*CORT, CENPS*	3.620	<0.001	
cg09495977	4:8271507	*HTRA3*	−2.420	0.017	
cg12333095	12:110437035	*ANKRD13A*	−3.485	0.001	
cg13818573	17:43045372	*C1QL1*	−3.596	<0.001	
cg21589280	1:85930152	*DDAH1*	−3.063	0.003	
cg03080985	6:80340683	*SH3BGRL2*	−3.077	0.003	
cg04330449	5:134871166	*NEUROG1*	−2.646	0.009	
cg05307923	10:1779667	*ADARB2*	−2.577	0.011	
cg08724517	4:156298205	*MAP9*	2.951	0.004	
cg11665562	14:90723462	*PSMC1*	−3.250	0.001	
cg14269514	1:151736130	*OAZ3, MRPL9*	−3.112	0.002	
cg24322623	11:17740431	*MYOD1*	−2.964	0.004	
cg27254601	16:29817104	*MAZ, BOLA2*	3.598	0.0005	[39]
cg15607672	14:57277228	*OTX2*	2.123	0.0363	

^a^ Position based on GRCh37/hg19 build. DNAm: DNA methylation; ICS: inhaled corticosteroid.

**Table 3:** Summary of the main findings of the only epigenomic study focused on the association of circulating miRNA with inhaled corticosteroid (ICS) response in childhood asthma [49].

miRNA	Odds Ratio	*p*-value
miR-206	0.60	0.004
miR-146b-5p	0.66	0.007
miR-222-3p	0.70	0.02
miR-409-3p	0.73	0.02
miR-223-5p	0.62	0.02
miR-126-5p	0.68	0.03
miR-339-3p	0.72	0.03
miR-30e-3p	0.70	0.03
miR-126-3p	0.74	0.03
miR-342-3p	0.80	0.04
miR-454-3p	0.77	0.04
miR-720	0.71	0.046

miRNA: micro ribonucleic acid.

## Supplementary Materials

Supplementary materials can be found at https://www.mdpi.com/1422-0067/21/8/2908/s1.

## Abbreviations

16S rRNA	16S ribosomal RNA
95% CI	95% confidence interval
ACQ	Asthma control questionnaire
AUC	Area under the receiver characteristics curve
BAMSE	Europeans from the Children, Allergy, Milieu, Stockholm, Epidemiology
BDR	Bronchodilator response
CAMP	Childhood Asthma Management Program
DNA	Deoxyribonucleic acid
EWAS	Epigenomic-wide association study
FDR	False discovery rate
FEV_1_	Forced expiratory volume in one second
FVC	Forced vital capacity
GACRS	Genetic Epidemiology of Asthma in Costa Rica Study
GALA	Genes-Environment and Admixture in Latino Americans
GWAS	Genome-wide association study
ICS	Inhaled corticosteroid
IgE	Immunoglobulin E
LABA	Long-acting beta agonist
LD	Linkage disequilibrium
LTRA	Leukotriene receptor antagonist
miRNA	Micro ribonucleic acid
NGS	Next-generation sequencing
OCS	Oral corticosteroid
OR	Odds ratio
PBCs	Peripheral blood cells
PBMCs	Peripheral blood mononuclear cells
PiCA	Pharmacogenomics in Childhood Asthma
RNA	Ribonucleic acid
RNA-seq	RNA sequencing
SAGE	Study of African Americans, Asthma, Genes and Environments
SABA	Short-acting beta agonist
SE	Standard error
SNP	Single nucleotide polymorphism
SRE	Steroid Responsiveness Endophenotype
TF	Transcription factor
WGCNA	Weighted gene co-expression network analysis
WGS	Whole-genome sequencing
